# Effect of 6 months’ flash glucose monitoring in adolescents and young adults with type 1 diabetes and suboptimal glycaemic control: managing diabetes in a ‘flash’ randomised controlled trial protocol

**DOI:** 10.1186/s12902-019-0378-z

**Published:** 2019-05-20

**Authors:** Sara E. Boucher, Andrew R. Gray, Martin de Bock, Esko J. Wiltshire, Barbara C. Galland, Paul A. Tomlinson, Jenny Rayns, Karen E. MacKenzie, Benjamin J. Wheeler

**Affiliations:** 1 Department of Women’s and Children’s Health, Dunedin School of Medicine, University of Otago, Dunedin, New Zealand; 2 Centre for Biostatistics, Division of Health Sciences, University of Otago, Dunedin, New Zealand; 3 Department of Paediatrics, University of Otago, Christchurch, New Zealand; 4 Paediatric Department, Canterbury District Health Board, Christchurch, New Zealand; 5 Department of Paediatrics and Child Health, University of Otago Wellington, Wellington, New Zealand; 6 Paediatric Department, Capital and Coast District Health Board, Wellington, New Zealand; 7 Paediatric Department, Southern District Health Board, Invercargill, New Zealand; 8 Endocrinology Department, Southern District Health Board, Dunedin, New Zealand; 9 Paediatric Department, Southern District Health Board, Dunedin, New Zealand

**Keywords:** Adolescents, Flash glucose monitoring, FreeStyle libre, Glucose monitoring, Glycaemic control, Intermittent continuous glucose monitoring, Self-monitoring of blood glucose, Type 1 diabetes, Young adults

## Abstract

**Background:**

Teenagers and young adults with type 1 diabetes (T1D) experience significant burden managing this serious chronic condition and glycaemic control is at its unhealthiest during this life stage. Flash glucose monitoring (FGM) is a new technology that reduces the burden of glucose monitoring by easily and discreetly displaying glucose information when an interstitial glucose sensor worn on the upper arm is scanned with a handheld reader, as opposed to traditional capillary glucose sampling by finger prick (otherwise known as self-monitored blood glucose, SMBG). The effectiveness of this technology and impacts of its long-term use in youth with pre-existing suboptimal glycaemic control are unknown. This study therefore aims to investigate the effectiveness of FGM in addition to standard care in young people with T1D.

**Methods:**

This is a two phase study programme including a multi-centre randomised, parallel-group study consisting of a 6-month comparison between SMBG and FGM, with an additional 6-month continuation phase. We will enrol adolescents with T1D aged 13–20 years (inclusive), with suboptimal glycaemic control (mean glycated haemoglobin (HbA1c) in past 6 months ≥75 mmol/mol [≥9%]). Participants will be randomly allocated (1:1) to FGM (FreeStyle Libre; intervention group) or to continue SMBG with capillary blood glucose testing (usual care group). All participants will continue other aspects of standard care with the study only providing the FreeStyle Libre. At 6 months, the control group will cross over to the intervention. The primary outcome is the between group difference in changes in HbA1c at 6 months. Additional outcomes include a range of psychosocial and health economic measures as well as FGM acceptability.

**Discussion:**

>If improvements are found, this will further encourage steps towards integrating FGM into regular diabetes care for youth with unhealthy glycaemic control, with the expectation it will reduce daily diabetes management burden and improve short- and long-term health outcomes in this high-risk group.

**Trial registration:**

This trial was registered with the Australian New Zealand Clinical Trials Registry on 5 March 2018 (ACTRN12618000320257p) and the World Health Organization International Clinical Trials Registry Platform (Universal Trial Number U1111-1205-5784).

## Background

Type 1 diabetes (T1D) is a serious chronic metabolic disorder often diagnosed during childhood and is characterised by high blood glucose levels resulting from pancreatic β-cell destruction [1]. Recently, the International Diabetes Federation estimated 500,000 children aged 14 years and younger had type 1 diabetes worldwide [2]. Increasing incidence [3–6] and prevalence [4, 6, 7] of T1D among youth has been observed in several countries. Complications of T1D include potentially life-threatening episodes of diabetic ketoacidosis (DKA) and severe hypoglycaemia, micro- and macrovascular diabetes-specific complications, and poorer mental health among children and adolescents with diabetes compared to those without diabetes [8]. There is no cure for T1D, therefore the goal of treatment is to achieve and maintain optimal glucose levels, particularly through insulin therapy, physical activity and diet [9].

Up until now, frequent daily self-monitored blood glucose (SMBG) testing has been essential for monitoring blood glucose levels, safety, and informing treatment decisions [10]. The evidence for the benefits of self-monitoring glucose levels on glycaemic control is well established [11, 12], with an association with lower glycated haemoglobin (HbA1c; the current standard measure of glycaemic control) by 0.5% for each additional SMBG check per day, up to 5 checks/day (*p* < 0.001) seen in an observational study of 20,555 children and adults with T1D [11]. Finger pricking (capillary glucose testing) is the most common approach to SMBG. However, pain, inconvenience, fear of stigmatization and embarrassment are common barriers to SMBG adherence among adolescents [13–17].

### Type 1 diabetes during adolescence

Adolescence is a high-risk period where glycaemic control is at its worst [18], treatment adherence is a particular challenge [19] and the strain of self-management takes a toll on adolescents’ and their parents’ quality of life [20]. Type 1 diabetes exchange data indicate only a minority of young people meet international guidelines for glycaemic control (HbA1c < 58 mmol/mol [< 7.5%]) [21]. During this life stage, barriers to treatment adherence include the major physical and cognitive changes and increasing independence regarding eating behaviour, physical activity and other aspects of lifestyle, such as sleep, increase the burden of managing T1D [22]. Most adolescents with T1D do not adhere to SMBG recommendations [19], with some adolescents undertaking SMBG only when experiencing symptoms of low or high blood glucose levels. Frequent misreporting and fabrication of SMBG data has also been described [23, 24].

### Flash glucose monitoring

Flash glucose monitoring (FGM) technology, sometimes referred to as intermittent continuous glucose monitoring (iCGM), is an accurate, safe, and acceptable approach to monitoring interstitial glucose levels in children (≥4 years) and adults [25–27]. FGM provides an up-to-date interstitial glucose level, a graph of retrospective data and predicted glucose trend when the user scans the sensor with a handheld receiver [26]. These interstitial glucose measurements have been found to be accurate compared with capillary blood glucose reference values [26]. Higher rates of scanning are associated with indicators of better glycaemic control, such as increased time in range (defined as glucose levels between 3.9 and 10.0 mmol/L [70–180 mg/dL]), reduced time in hyperglycaemia (> 10 mmol/L [> 180 mg/dL]), and improved HbA1c [28, 29]. FGM is less expensive than a similar technology, traditional continuous glucose monitoring (CGM), and does not require regular calibration, like the majority of current CGM systems. However, FGM is retrospective, and therefore does not have the CGM feature of automatic alerts in response to pre-defined low or high glucose levels. Adverse events associated with FGM use are typically limited to skin issues associated with sensor insertion and reactions to sensor adhesive [18, 25, 27, 30].

Evidence of the potential to increase glucose monitoring among adolescents by using FGM technology is emerging [29–32]. FGM may provide an important opportunity to engage adolescents in their diabetes care by reducing their disease burden and facilitating access to glucose data more frequently, which in turns enables adolescents to make better informed management decisions. The easy instant access to glucose levels that FGM affords is particularly suited for young people where motivation is lacking and the burden of disease is high [33]. However, to our knowledge, there is no evidence demonstrating the superiority of FGM over SMBG for improving clinical outcomes in this challenging patient population. A 6-month randomized controlled trial evaluating FGM among adults with well controlled T1D reported reduced time in hypoglycaemia in the intervention group compared to the SMBG group, but no significant change in HbA1c [31]. Short-term improvements in glycaemic outcomes, including time in range and reduced time in hyperglycaemia, as well as improvements in HbA1c, have been observed in a single-arm study among children and teenagers using FGM for 8 weeks [30]. There is evidence of clinically significant improvements in HbA1c (by ≥0.5% in HbA1c) among T1D patients aged 1–25 years being sustained at 12 months [32] in a real-world setting. This finding is similar to some other diabetes technology, with a recent insulin pump study among adolescents with unhealthy glycaemic control showing sustained improvements in HbA1c from baseline out to 12 months [34]. The same study reported short-term improvements in diabetes-specific and generic health- related quality of life (HRQoL) related to insulin pump use that were not sustained [34]. Research is clearly warranted to investigate the effectiveness of FGM on glycaemic control among young people, particularly those with the unhealthiest glycaemic control (who are usually excluded from manufacturer-funded studies and are plausibly the group with the greatest potential to benefit if FGM is effective). Broader impacts of FGM on salient outcomes such as fear of hypoglycaemia, diabetes treatment satisfaction, and HRQoL also warrant investigation.

### Aim, objectives and hypotheses

The overall study aim is to evaluate, in a randomised controlled trial, the clinical effectiveness of FGM compared to SMBG for adolescents with T1D and a history of suboptimal glycaemic control. The primary objective is to investigate the effectiveness of FGM compared to SMBG in reducing HbA1c (i.e., the primary analysis will be on a modified intention to treat basis, using all available data).

The secondary objectives are to investigate:

1. the effectiveness of FGM compared to SMBG in increasing glucose monitoring behaviour during the 6-month trial period;
2. the effectiveness of FGM compared to SMBG in changing disease specific and generic HRQoL, fear of hypoglycaemia, and treatment satisfaction during the 6-month trial period;
3. acceptability and feasibility of using FGM to self-monitor glucose levels during the 6-month trial period; and
4. Adverse events (e.g. skin problems, diabetic ketoacidosis, severe hypoglycaemia) during the 6-month trial period.

The tertiary objectives are to explore:

1. all of the primary and secondary analyses using participants from the FGM group who scanned at least once per day 12 days out of 14 in the two weeks prior to the 6 month study visit (which is a definition modified from a recent landmark CGM study [35]) and excluding any participants from the SMBG group who used FGM at any time during the study (i.e., a per protocol analysis with potential confounding by characteristics associated with compliance, although all participants in the study have a recent history of suboptimal glycaemic control and so are likely to have similarly low baseline rates of SMB testing)
2. the impact of FGM compared to SMBG on sleep and physical activity during the 6-month trial period; and
3. all study outcomes in both groups at 12 months, including estimating within-group changes in the FGM group from 6 months to 12 months (to investigate persistence of changes) and within-group changes in the crossed over group from 6 months to 12 months (to see if any such changes are consistent with those in the FGM group from baseline to 6 months).

It is hypothesized that compared to SMBG, FGM will result in a clinically relevant (at least 1.0%; 10 mmol/mol) and statistically significant reduction in mean HbA1c at 6 months’ post-baseline. Additionally, it is hypothesized that there will be statistically significant improvements in other important outcome measures in health, wellbeing, and diabetes management in the FGM intervention group at 6 months, compared to the SMBG group.

## Methods

### Study design

**Fig. 1:**
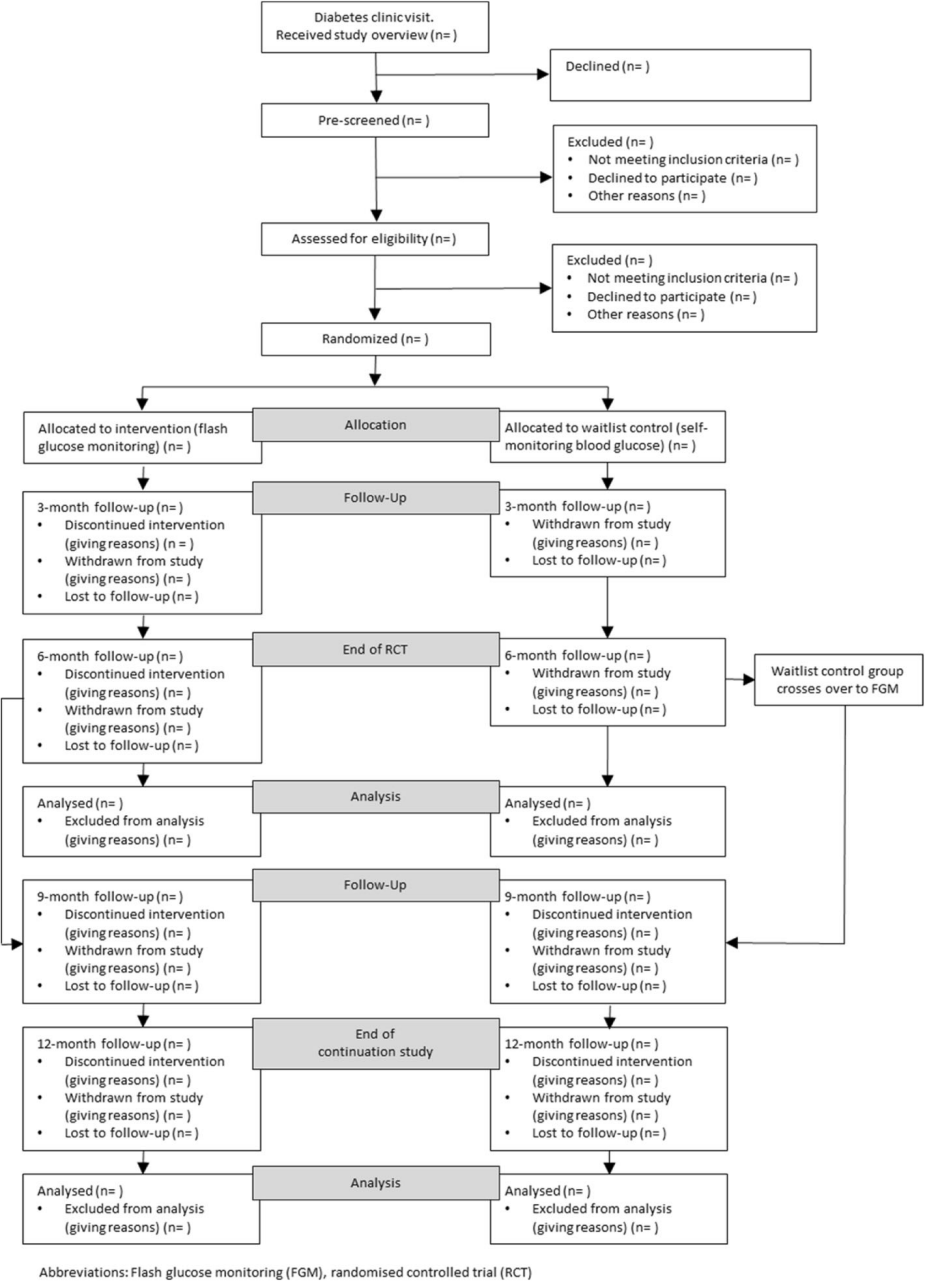
CONSORT flow diagram

Managing Diabetes is a ‘Flash’ is a two phase study programme consisting of a multi-centre randomised, parallel-group study consisting of a 6-month comparison between SMBG and FGM, including a 6-month continuation phase (see Fig. 1). Once enrolled, the total study period is 12 months for each participant (12 months of FGM for the intervention group will enable an investigation of sustained FGM outcomes). During the continuation phase, the FGM group will continue the intervention for a further 6 months and the control group will cross over and receive a 6-month FGM intervention. The primary purpose of offering FGM to the control group at 6 months is to encourage retention and engagement.

Scientific peer review was undertaken both internally, by the Department of Women’s and Children’s Health, University of Otago, and externally, by the Cure Kids Medical and Scientific Advisory Committee. The Southern Health and Disability Ethics Committee approved this study (17/STH/240). The protocol underwent Māori (indigenous New Zealanders) consultation, which fostered input into this study. All district health boards approved recruitment and conduct of the study at their site. The trial was officially registered with the Australian New Zealand Clinical Trials Registry (ACTRN12618000320257p; http://www.ANZCTR.org.au/ACTRN12618000320257p.aspx) on 5 March 2018 and was issued a Universal Trial Number (U1111–1205-5784) by the World Health Organization International Clinical Trials Registry Platform. The University of Otago is assuming overall responsibility for the initiation and management of the trial (i.e., is the Trial Sponsor). The study is in no way funded or affiliated with the manufacturer of the FGM system under investigation or any manufacturer of a competing system.

### Recruitment

Adolescents with T1D meeting eligibility criteria for age, duration since diabetes diagnosis, and absent CGM/FGM use in the previous 4 months will be invited to participate in the study during routine diabetes clinic visits. The diabetes clinics were selected where an investigator was affiliated with both the University of Otago Medical School and a district health board (New Zealand’s regional organisations responsible for delivering publicly funded healthcare services to both urban and rural patients). Combined, these district health boards (DHBs) serve a population of approximately 1.2 million people [36] and provide care to approximately 619 people aged 13–20 with T1D [37]. It is assumed all patients with diabetes in these catchments would attend these clinics, as minimal private practice in diabetes is provided within New Zealand’s predominantly public health system.

After a potential participant is identified by diabetes clinic staff, they will receive a brief overview of the study and their interest in the study will be ascertained. Patients who are interested in the study will then be pre-screened over the phone by research staff and sent an information sheet, which explains the study objectives and participant responsibilities in full. Research staff will respond to all study-related questions and schedule a screening visit to confirm eligibility. The inclusion and exclusion criteria for assessing eligibility to participate in the study (Table 1) will be used during a screening visit conducted with the potential participant, any accompanying participant support people, family, or whānau (extended family) and research staff. Each centre will pre-screen and refer patients until the target sample size is achieved. Trial investigators will not receive financial or non-financial incentives for enrolment.

**Table 1:** Inclusion and exclusion criteria for participation in Managing Diabetes in a ‘Flash’

Inclusion criteria	Exclusion criteria
• Aged 13 to 20 years (inclusive)	• Any severe diabetes related complications (nephropathy on treatment, retinopathy with associated visual loss – milder degrees will not be excluded)
• Mean HbA1c over previous 6 months ≥75 mmol/mol (≥9%)	
• Diagnosed with T1D for at least 12 months	• Other severe uncontrolled medical or psychiatric co-morbidity/severe mental illness
• Prescribed > 0.5 units of insulin/kg/day (with no restrictions based on insulin regimen)	
• Planned to continue with routine clinical care during the initial 6-month RCT	• Currently using a CGM or FGM device or has used one continuously (other than for intermittent hospital use) within the previous 4 months
• Resident in and expecting to remain in regions affiliated with the Canterbury, Capital and Coast, and Southern District Health Boards for the following year	• Participation in another device or drug study that could affect glucose measurements during the study period
	• Pregnant, lactating, or plan to become pregnant
• Ability to understand study procedures, including English language proficiency, and to comply with them for the entire length of the study	
	• Inability of individual (for those aged 16 years and older) or legal guardian (for those aged 13–15 years) to give written informed consent

### Sample size

Based on our data from an earlier study in this population, the standard deviation of HbA1c amongst those with values 75 mmol/mol (≥9%) or greater (and irrespective of self-monitoring blood glucose frequency) was 18.6 mmol/mol [38, 39]. Assuming a correlation between repeated measures of 0.7, a sample size of 58 (29 per group) would provide 80% power to detect a difference in mean changes for HbA1c of 1% (10.93 mmol/mol) using a two-sided test at the 0.05 level. This would be considered a clinically important difference, of a similar magnitude to other proven technologies such as insulin pumps or CGM [40]. To account for a small amount of missing data and loss to follow-up (anticipated to be < 10%), we will recruit a sample size of 64 (32 participants per group) at baseline. Reasons for non-retention of participants (i.e., consent withdrawn, moving outside of the study DHBs, or lost to follow-up) will be recorded and shown in a CONSORT flow diagram (Fig. 1).

### Study procedures

#### Screening and enrolment

After being given an opportunity to review the participant information sheet and consider whether they want to be involved in the study, and prior to screening, adolescents and young adults aged 16–20 years and a parent/guardian of adolescents aged 13–15 years must provide written informed consent for their participation in the study. Parents/guardians will also be asked for their consent to provide information about themselves. Adolescents aged 13–15 years must also provide written assent for their participation. A point of care (POC; DCA Vantage Analyzer, Siemens Healthcare Diagnostics, Ireland) HbA1c will be measured when POC HbA1c has not been assessed in the previous 14 days. All female patients will provide a urine sample and study staff will follow the manufacturer’s guidelines for conducting the pregnancy test (EasyCheck® Pregnancy Test, Hangzhou Clongene Biotech Co, Ltd., China). Date of diabetes diagnosis (for subsequent calculation of duration of diabetes), current insulin regimen, insulin dosing, HbA1c measurements in previous 6 months, and co-morbidities will be recorded from electronic medical records. Diabetic ketoacidosis in the past 6 months, and severe hypoglycaemia events (defined as requiring the assistance of another person to treat [41]) in the past 6 months will also be recorded from electronic medical records to provide baseline estimates of frequency for these events. Any patient can withdraw (or be withdrawn by their parent or guardian for those aged 13–15) from the study at any point and return to their usual medical care.

#### Randomisation

Patients who give consent for participation and fulfil the eligibility criteria will be enrolled in the study and randomly allocated by an offsite biostatistician (ARG) in batches using a 1:1 ratio to either the waitlist control (SMBG) group or the intervention (FGM) group. The statistician will be blinded to allocation arm and will use non-informative group codes until all planned analyses are completed. As gender and pre-study HbA1c may significantly affect the primary outcomes, minimisation will be used (based on gender [male, female; HbA1c [75 to < 100 mmol/mol, ≥ 100 mmol/mol; 9.0 to < 11.3%, ≥ 11.3%]) and with a small random component (20%) along with randomly ordering the participants in each batch used to preserve allocation concealment. The study group will be revealed to the participant at the baseline visit after all assessments have been completed.

#### Study groups

##### Usual care (waitlist control) group

Participants allocated to the waitlist control group will receive standard diabetes care from their usual provider. In New Zealand, these clinics are attended regularly (usually a minimum of every 3 months) to provide diabetes care by a multi-disciplinary team (i.e., paediatric or adult endocrinologist/diabetologist (depending on patient age), diabetes nurse specialist, dietician, psychologist/social worker). Participants will continue self-monitoring their blood glucose levels with their usual glucose meter per recommendations from their clinical care team. All other aspects of diabetes care will remain with their usual clinical care facility and will be in no way altered by the study team. No additional diabetes care, support, or advice will be provided by the study team making the provision of the FreeStyle Libre (Abbot Diabetes Care, Witney, Oxon, UK) FGM system, along with instruction in its use, the only difference between intervention groups. To maximise study retention in the control group, participants allocated to this group will receive FGM for 6-months at the completion of their 6-month follow-up study visit.

##### Flash glucose monitoring (intervention) group

Participants randomized to the intervention group will continue routine care (as described above) and receive a FGM system, which includes a reader, USB cable, power adapter, user’s manual, and quick start guide. In addition, the FGM group will be provided and talked through a 1-page single-sided handout summarising key educational information from the manufacturer’s user manual, instructions to confirm their blood glucose level before therapeutic interventions or corrective action if hypoglycaemic or hyperglycaemic glucose levels or symptoms occur, recommendations to prolong sensor life, and instructions to report adverse events (see Supplementary material). The first FreeStyle Libre sensor will be applied by trained staff (i.e. diabetes nurse or research staff trained by a FreeStyle Libre distributor territory manager) at the baseline visit using the manufacturer’s quick start guide. Participants will receive a spare sensor in case of sensor failure before the follow-up visit and a “patch” made from pre-cut kinesiology tape (FreeStyle Libre patch, Rockadex, Perth, Australia) to apply over the sensor in the event the sensor is at risk of falling off prematurely (i.e., due to impaired adhesion to skin or sensor adhesion to the adhesive applied to the skin). An alternative product (Hypafix®, BSN medical GmbH, Hamburg, Germany) will be provided to participants with a known skin sensitivity to Rockadex.

Participants will return in 14 days for the sensor site to be inspected for skin problems, apply the next sensor under supervision, and receive a 3-month FGM supply (i.e., 6 sensors and Rockadex or Hypafix patches). At this 14-day follow-up visit, staff will briefly provide more advanced FGM education on the trend arrows (specifically, the rate of change associated with each arrow direction and arrows indicating the need for a blood glucose check), the target glucose range graph, and instructions to review glucose readings on a computer using a 2-page handout (see Supplementary material). In exceptional circumstances, such as participants living more than 3 h from a clinic, the participant will apply their first sensor under trained staff supervision, receive a 3-month sensor supply, and will be followed-up in 14-days via teleconference software (Zoom, Zoom Video Communications, San Jose, CA, USA). Participants will apply their sensor unsupervised every 2 weeks for the remainder of the study. No further FGM education will be provided and no adjustments to treatment will be made by study staff. Furthermore, study staff will not interfere with participants’ adherence to flash glucose monitoring. Participants will be instructed to seek guidance on scanning frequency from their lead diabetes care provider.

Additional FGM supplies will be provided at the 3-, 6-, and 9-month follow-up visits. During all follow-up visits, retrospective glucose readings will be downloaded from the FGM reader for the previous 2 weeks, and reviewed for glucose levels below 4.0 mmol/mol between the hours of 10 pm and 7 am. All events of nocturnal hypoglycaemia in the previous 2 weeks will be reported to the appropriate diabetes care provider for follow-up.

### Procedures

The baseline measurements will be collected approximately 7 days from the screening visit. Follow-up assessment visits will be scheduled within a 14-day window, defined as 7 days before and 7 days after the due date. Assessment visits will be undertaken by data collectors trained centrally on the study requirements and measurement procedures. Blinding participants and research staff to group allocation will not be feasible. Participants will receive a $20 gift voucher in recognition of participation at each of the baseline and follow-up assessment visits.

### Outcome measures

The primary outcome measure is the difference in change in HbA1c between groups at 6-months.

All other measures are for secondary and tertiary (exploratory) outcomes. The timing of all assessments is presented in Table 2.

**Table 2:** Managing Diabetes in a ‘Flash’ schedule of assessments

	Time point				
Outcome	Baseline	3 m	6 m	9 m^a^	12 m^a^
Adverse events					
Cutaneous, diabetic ketoacidosis, severe hypoglycaemia	Every 2 weeks from baseline				
Device performance					
FGM/SMBG device malfunctions, FGM sensor failures	Every 2 weeks from baseline				
Clinical					
Glycaemic control	X	X	X	X	X
Height	X	X	X	X	X
Weight	X	X	X	X	X
Glucose monitoring behaviour					
Blood glucose monitoring	X	X	X	X	X
Interstitial glucose monitoring		X	X	X	X
Habitual sleep and physical activity					
Sleep	X	X	X		
Physical activity	X	X	X		
Participant reported					
Diabetes treatment satisfaction	X	X	X		X
Fear of hypoglycaemia	X	X	X		X
Flash glucose monitoring acceptability		X	X		X
Health-related quality of life (generic)	X	X	X		X
Health-related quality of life (diabetes-specific)	X	X	X		X
Sleep quality and quantity	X	X	X		X

^a^ Denotes continuation phase of study

Abbreviations: *FGM* Flash glucose monitoring, *SMBG* self-monitoring blood glucose

### Adverse events and glucose monitoring device performance

All participants will be sent a link to an electronic questionnaire fortnightly, via both text and email, to report any episodes of cutaneous problems (e.g., infection, itching, rash, pain, subcutaneous haemorrhage), severe hypoglycaemia (i.e., child experiences altered mental status and as a result is unable to assist in their care, or is semiconscious or unconscious) [41], diabetic ketoacidosis (DKA), or missing school/work due to their diabetes. Up to four contact attempts will be made to non-responders. Data from safety surveys will be reviewed by research staff upon receipt by the study team. Participants will be referred to their general practitioner, diabetes team, or emergency department, as appropriate, for management of medical events. Adverse events (events which result in harm to a participant) will be reported to an internal safety monitoring committee made up of co-investigators with a clinical background and expertise in trials and diabetes.

The adverse events questionnaire will also collect data on FGM performance (i.e., sensor problems, reader problems), FGM adherence (i.e., duration of time each sensor is worn) and blood glucose meter performance (i.e., device malfunctions).

### Clinical outcomes

#### Anthropometry

Trained staff members will measure adolescents’ weight and height using standard procedures and calibrated instruments. Weight will be measured once with a fixed scale (DigiTol, Toledo, Switzerland or similar) or portable scale (Tanita Corporation, Japan or similar) to the nearest 0.1 kg, with shoes and heavy clothing removed. Height will be measured once to the nearest 0.1 cm using a fixed stadiometer (Harpenden stadiometer, Holtain Limited, Pembs, UK or similar) or a portable stadiometer (Leicester Height Measure, Invicta Plastics Ltd., Oadby, England). Portable devices will be used when measurements are conducted outside the clinic (participants’ home or a community centre). Height and weight will be used to calculate body mass index (BMI)- z-scores using Centers for Disease Control and Prevention growth standards [42].

#### Demographics

At the screening visit, a self-administered questionnaire will collect demographic information including age, gender ethnicity, address, and education level. Participants may choose to select more than one ethnicity; however, each person will be allocated to a single ethnic group for the purposes of statistical analyses that will be prioritised in the order of Māori, Pacific, Asian and European/Other [43]. The address where the participant lives more than 50% of the time will be used to assess their NZDep2013 deprivation score, which is a validated index of the relative socioeconomic deprivation of the area in which an individual lives [44].

#### Glycaemic control

Glycated haemoglobin (HbA1c) will be measured by traditionally calibrated point-of-care instruments (DCA Vantage Analyzer, Siemens Healthcare Diagnostics, Ireland), which meets acceptance criteria for HbA1c [45]. Measurements > 130 mmol/mol (maximum reading possible) will be recorded as 130.

#### Glucose monitoring behaviour

##### Self-monitoring blood glucose

The mean blood glucose level and total number of blood glucose tests will be downloaded into SmartLog (SmartLog Diabetes Management Software version 2.4.4, i-SENS, Inc., Seoul, Korea) from the currently funded blood glucose meter (CareSENS dual, i-SENS, Inc., Seoul, Korea) for the previous 2 weeks and recorded. Insulin pump data (if participant using a pump) will be downloaded using diasend® (Diasend/Glooko, Goteborg, Sweden) for the previous 2 weeks, and the mean blood glucose level and the mean number of tests recorded.

#### Flash glucose monitoring

All data will be downloaded at every follow-up visit and exported from the FGM reader using the FreeStyle Libre computer software version 1.0 (Abbott Diabetes Care, Ltd., Witney, Oxon, UK). Individual glucose measures, mean interstitial glucose level, and frequency of scans in the previous two weeks will be recorded.

#### Health status and quality of life

Validated instruments will assess the self-reported impact of FGM on adolescents’ health and quality of life at baseline, 3-, 6-, and 12-months. These instruments have been widely used in research and have demonstrated reliability and validity in this population. Data will be collected via electronic (REDCap; Research Electronic Data Capture) self-administered questionnaires or paper questionnaires prior to clinical assessments and the order of administration will be standardized to increase reliability. REDCap [46] is a secure, web-based application designed to support data capture for research studies. Together the questionnaires will take between 30 and 45 min to complete. All questionnaires are administered in English. Participant reported outcomes including overall health-related generic quality of life, health-related diabetes-specific quality of life, fear of hypoglycaemia, diabetes treatment satisfaction, and sleep outcomes will be monitored during the study and clinical care teams will be notified if participants report physical or mental problems necessitating follow-up.

1. PedsQL™ generic core scale

The Paediatric Quality of Life Inventory (PedsQL™ 4.0) Generic Core Scale [47] is a 23-item measure of four dimensions related to an adolescents’ health-related quality of life (physical functioning, emotional functioning, social functioning, school functioning) in the past month. Scores are reported on a scale from 0 to 100 with higher scores indicating better health-related quality of life. Internal consistency reliability has been shown to be acceptable for the Total Scale Score (alpha = 0.88), Physical Health Summary Score (alpha = 0.80), and Psychosocial Health Summary Score (alpha = 0.83). [48] The PedsQL™ 4.0 has been shown to distinguish between children and adolescents with diabetes and children and adolescents without diabetes [49].

2. PedsQL™ diabetes module

The Paediatric Quality of Life Inventory Diabetes Module for Type 1 diabetes (PedsQL™ Version 3.0 Type 1 Diabetes module) [49] is a 28-item self-report measure of T1D specific health-related quality of life in adolescents in the past month. The scoring is identical to the PedsQL 4.0, with higher scores indicating fewer symptoms or problems. In a sample of children and adolescents with T1D and type 2 diabetes, the mean alpha-coefficient for the Diabetes Module scales was acceptable (alpha = 0.71, range: alpha = 0.63 to 0.81) and the Diabetes Module demonstrated inter-correlations with dimension of generic and diabetes-specific HRQOL [49].

3. Fear of hypoglycaemia

Participants aged 18–20 years, inclusive, will complete the adult Hypoglycaemia Fear Survey-II (HFS-II) [50, 51], which is a 33-item measure of fear of hypoglycaemia (behaviour subscale) and worries related to various aspects of hypoglycaemia (worry subscale) in the past 6 months. All items are summed to obtain a Total Score (score range: 0–132; higher Total Scores reflect a greater fear of hypoglycaemia) and each set of items for the two subscales are summed to yield a Behaviour Score (score range: 0–60; a higher score reflects a greater tendency to avoid hypoglycaemia and/or its negative consequences) and a Worry Score (range score: 0–72; a higher score indicates more worry concerning episodes of hypoglycaemia and its consequences). These sums will be divided by the total number of items in each scale/subscale to obtain an item mean score. The Hypoglycaemia Fear Survey for Children (HFSC) [52] is a 25-item instrument adapted from the adult HFS. The HFSC will be completed by adolescents in the study aged 13–17 years, inclusive.

Overall, higher scores reflect greater fear of hypoglycaemia, a higher score on the Behaviour Subscale reflects a greater tendency to avoid hypoglycaemia and/or its negative consequences, and a higher score on the Worry Subscale indicates more worry concerning episodes of hypoglycaemia and its consequences. The CHFS has shown adequate internal consistency (HFSC behaviour subscale alpha = 0.70; CHFS worry subscale alpha = 0.89; and CHFS-Total alpha = 0.85) [52]. HFSC worry subscale and total scores have been shown to correlate significantly with other measures of anxiety. [52] HFS-II and CHFS total scores and subscale scores will be calculated as z-scores standardised to the instrument-specific and baseline means and standard deviations.

4. Diabetes treatment satisfaction

The Diabetes Treatment Satisfaction Questionnaire- status version (DTSQs) [53] is a 12-item measure of a patient’s current treatment satisfaction that includes subscales to measure diabetes treatment satisfaction and perceived diabetes control. The Diabetes Treatment Satisfaction Questionnaire-change version (DTSQc) is a 12-item self-report measure of change in a patient’s satisfaction with their diabetes treatment regimen, which was developed to overcome potential ceiling effects (i.e., where respondents score maximum or near-maximum satisfaction at baseline and can show little or no improvement at follow-up).

#### Flash glucose monitoring acceptability

Adolescents in the FGM group will evaluate the FreeStyle Libre FGM system acceptability at 3- and 6-months, and all participants will answer acceptability questions at 12-months using a non-validated instrument adapted from previous similar research [25]. On an ordinal scale from 0 (strongly disagree) to 5 (strongly agree), participants will rate their opinion in regards to the following areas: acceptability of sensor application, wear/use of the device and comparison to SMBG.

#### Sleep and physical activity

##### Accelerometry

Adolescents will wear an accelerometer (ActiGraph, wGT3X-BT, Pensacola, FL, USA) on the non-dominant wrist for 7 days and 8 nights (continuously), except during activities involving water (e.g., showering and swimming) prior to the baseline and 3- and 6-month follow-up visits. The ActiGraph wGTX3- BBT is a motion sensor worn like a wrist-watch that gives objective information about physical activity and sleep, including sleep disturbance. ActiGraphs will be initialized using 15 s epochs. Data will be scored using the count-scaled algorithm developed within our lab for scoring sleep and physical activity in MATLAB (MathWorks, Natick, MA, USA) [54, 55]. Standard sleep variables from the actigraphy outputs will be calculated [56]: sleep onset, sleep offset; sleep duration (sleep period time and total sleep time); wake after sleep onset; and sleep efficiency. Given the lack of validated cut-offs for determining intensity of activity spanning adolescents and young adults, activity accelerometry will be measured as counts per minute. The data will enable an exploratory analysis to investigate the association between wearing the FreeStyle Libre and changes in activity.

##### Pittsburgh sleep quality index

Habitual sleep will be measured at baseline, 3- and 6-months using the Pittsburgh Sleep Quality Index (PSQI) [57]. The PSQI is a 19-item self-report measure of subjective sleep quality and sleep quantity in the previous month. Originally devised as a 19-item questionnaire for adults, five questions related to sleeping with a bed partner will not be included. The PSQI generates 7 domains for subjective sleep quality, sleep latency, sleep duration, sleep efficiency, sleep disturbance, sleep medication, and daytime dysfunction, with each component score ranging from 0 to 3, and summed to produce a global score. A global score > 5 suggests a “poor sleeper” with significant sleep complaints. There is evidence of acceptable internal consistency (alpha = 0.77) with item-rest correlations ranging from 0.43 to 0.61 in adolescents [58].

#### Other measures

##### Parent/caregiver self-reported questionnaires

At baseline, parents of enrolled participants who provide written consent for their own participation in the study will complete a short questionnaire collecting demographic characteristics (e.g., age, gender, education level, and ethnicity). At baseline, 3-, 6-, and 12-months post-baseline, parents’ perceptions of their child’s wellbeing will be assessed by a self-report questionnaire. Parents/caregivers will also complete parent-proxy versions of questionnaires to assess their perceptions of their child’s quality of life [47, 49], their own fear of their child experiencing hypoglycaemia [52], and their own satisfaction with their child’s diabetes treatment [53]. The DTSQ and HFS are not available for parents of participants 18 years of age and older.

### Data management

All study participants will be assigned a non-informative study identification number. Data will be recorded and stored electronically in REDCap, which is hosted at the University of Otago. REDCap features will help ensure adherence to time-frames, compliance to measurement procedures, and completeness of data. Data will be routinely checked for missing and/or erroneous values by the study coordinator. Only research staff and investigators will have access to the electronic study records. The ethics committee did not require auditing for this study.

At the end of the study, original data collection sheets and written informed consent will be stored securely at the lead site along with copies of all data collected electronically. At the end of the study, the lead investigator will retain an electronic copy of the cleaned data set, with all identifying information removed.

### Statistical analysis

Means and standard deviations (SDs) will be reported for continuous variables (i.e., age, diabetes duration, HbA1c, glucose monitoring behaviour, questionnaire scores) that are approximately normally distributed, geometric means and SDs for those that are approximately log-normally distributed, and medians and IQRs otherwise. Categorical variables will be described as the number and percent of participants in each category (i.e., gender, ethnic group, insulin regimen). Adverse events will be categorised by the type of event (e.g., severe hypoglycaemia, DKA, sensor insertion issues, sensor wear issues). The frequency of events will be reported by event category.

A mixed linear model will be used to test the difference in HbA1c, with the primary outcome being at 6-months, between the intervention and control groups, using data from all time points with a random participant effect used to accommodate the repeated measures and a group-time interaction to model differences in longitudinal changes. Changes in number of glucose measurements performed and patient-reported outcome measures (i.e., PedsQL generic core scale, PedsQL diabetes module, HFS, and DTSQs scores) from baseline through to 6 months will be calculated by comparing scores from control and intervention group participants using Poisson (or negative binomial regression if there is evidence of over-dispersion) for count outcomes and linear mixed models for continuous outcomes with all collected data included. Model residuals will be examined along with the distribution of random effects. If residuals are positively skewed, natural logarithmic transformations will be investigated and retained if these improve the satisfaction of model assumptions. Mixed quantile regression will be investigated for continuous outcomes if issues with model residuals remain following such transformations. If HbA1c values of 130 are observed, mixed Tobit models with right censoring at 130 will be used for this outcome instead. All mixed models will use restricted maximum likelihood (REML) estimation for the variance components.

Some secondary analyses, such as those involving the wait-list control after they cross over, will only include participants from one group and so group (and group-time interaction) terms will not be included in these models.

The main analyses will follow a modified intention- to-treat principle with all participants analysed in the group to which they were randomised, regardless of actual sensor wear. Per protocol analyses will be also be investigated by including only those from the FGM group who scanned at least 12 out of 14 days in the two weeks prior to the 6-month study visit and excluding any participants from the SMBG group who used FGM at any time during the study. All models will include variables used in minimization [59].

Any missing HbA1c values will be imputed using multiple imputation with chained eqs. (100 imputations after at least 100 burn-ins) and with the imputation model including: gender, HbA1c status at baseline as well as values of that variable from other time points, insulin regimen, frequency of self-monitored glucose (capillary or interstitial) and socioeconomic status. If missing data exceeds 10%, plausible scenarios involving informative missing data will be investigated to explore the robustness of findings.

Statistical analyses will be performed using R 3.5.2 or Stata 15.1 software (or later versions) with two-sided *p* < 0.05 considered significant. To maintain the integrity of the study, statistical analyses will be performed by the biostatistician blinded to group allocation.

#### Post-trial care

Should this study provide evidence of the effectiveness of FGM, it will be ideal to provide ongoing funded access to FreeStyle Libre sensors to study participants and other young people with T1D (FGM is currently not publically funded within New Zealand). In preparation for this study, discussions have begun with PHARMAC (the New Zealand government agency that decides which medical devices to publicly fund in New Zealand) on publically-funded access to FGM.

## Discussion

Adolescence is a challenging time for diabetes management and adherence. Flash glucose monitoring technology may assist adolescents with self-monitoring glucose levels and inform treatment decisions that result in improved glycaemic control. *Managing Diabetes in a ‘Flash’* is a 12-month study that will combine a 6-month RCT, immediately followed by a 6-month observational continuation study. There are several strengths of the study, including: 1) being funded and conducted independent of the FreeStyle Libre manufacturer; 2) the RCT design, which is the gold standard for evaluating the effectiveness of interventions such as FGM; 3) the 6 month duration of the RCT and observational studies allowing for insight into effects following an initial “novel” period, 4) the waitlist control group who will access the intervention at 6-months, which may maximise study retention; 5) no additional diabetes support to the intervention group beyond providing the FGM system; and 6) the real-life context which will help gauge the effectiveness of FGM among adolescents with suboptimal glycaemic control. The observational phase enables the longest evaluation of FGM in this patient population to date, enabling research of whether or not outcomes at 6 months appear to be sustained at 12 months despite the lack of a control group for this comparison. Overall, the study outcomes are wide in scope and will provide a broad evaluation of the application of FGM among young people with T1D that provide valuable evidence to inform shared decision making between the patient, their family/families, the clinical team, and healthcare funders.

## Abbreviations

BMI: Body mass index
DHB: District health board
DKA: Diabetic ketoacidosis
DTSQc: Diabetes Treatment Satisfaction Questionnaire-status version
DTSQs: Diabetes Treatment Satisfaction Questionnaire-change version
FGM: Flash glucose monitoring
HbA1c: Glycated haemoglobin
HFSC: Hypoglycaemia Fear Survey for Children
HFS-II: Hypoglycaemia Fear Survey-II
IQR: Interquartile range
MVPA: Moderate-vigorous physical activity
PedsQL: Paediatric Quality of Life Inventory
PSQI: Pittsburgh Sleep Quality Index
REDCap: Research electronic data capture
REML: Restricted Maximum Likelihood (also Residual Maximum Likelihood)
SD: Standard deviation
SMBG: Self-monitoring blood glucose
T1D: Type 1 diabetes
